# Localized Discoid Lupus Erythematosus of the Auricular Concha: A Case Report Highlighting the Diagnostic Relevance of the Carpet Tack Sign

**DOI:** 10.7759/cureus.106742

**Published:** 2026-04-09

**Authors:** María Paula Muñoz, Cindy Lorena Caceres, Camilo Morales

**Affiliations:** 1 Dermatology, Centro Dermatologico Federico Lleras Acosta, Bogotá, COL; 2 Dermatology, Clinica Privada Dermatologia, Bogotá, COL

**Keywords:** chronic cutaneous lupus erythematosus, clinical signs, dermoscopy findings, discoid lupus erythematosus (dle), early diagnos

## Abstract

Discoid lupus erythematosus is a chronic autoimmune inflammatory dermatosis characterized by erythematous, scaly plaques that may progress to permanent atrophy, scarring, and pigmentary alterations. The auricle is a recognized site of involvement in discoid lupus erythematosus; however, isolated lesions confined to the auricular conchae are less frequently described and may pose diagnostic challenges because of their clinical resemblance to other inflammatory, infectious, or neoplastic conditions of the ear. We report the case of a 35-year-old woman with a 15-year history of asymptomatic bilateral lesions involving the auricular conchae. Physical examination revealed violaceous, poorly defined oval plaques with thick, adherent whitish scale, accompanied by multiple whitish filiform papules consistent with the carpet tack sign. Dermoscopic evaluation demonstrated a violaceous-brown plaque with thick adherent scale, multiple whitish punctate projections, and perifollicular whitish halos. Histopathological examination showed epidermal atrophy, prominent follicular plugging, interface dermatitis with apoptotic keratinocytes, dermal melanophages, thickening of the basal membrane, and increased interstitial mucin, findings consistent with discoid lupus erythematosus. This case highlights the diagnostic value of recognizing characteristic clinical and dermoscopic features, particularly the carpet tack sign, in localized auricular disease. Early identification enables timely diagnosis and appropriate management, thereby helping to prevent irreversible scarring and long-term sequelae in this anatomically and aesthetically sensitive area.

## Introduction

Discoid lupus erythematosus (DLE) is the most common form of chronic cutaneous lupus erythematosus. It is a chronic autoimmune inflammatory dermatosis that predominantly affects photoexposed areas and is characterized by erythematous, scaly plaques that may progress to atrophy, permanent scarring, and pigmentary alterations [1-3]. While head and neck involvement occurs in up to 80% of cases, isolated auricular presentation is considered uncommon. Nevertheless, this specific location represents a relevant challenge due to the risk of disfiguring scarring and the diagnostic complexity it poses in daily practice [1-3]. Histopathologically, DLE is defined by interface changes (inflammation at the dermoepidermal junction) and follicular plugging (keratin accumulation within hair follicles), which directly correlate with its clinical manifestations [4].

Clinically, auricular DLE presents as chronic erythematous plaques with adherent scale, sometimes demonstrating the "carpet tack sign," a descriptive term referring to keratotic follicular plugs attached to the undersurface of detached scales. It may exhibit recurrent erosions or crusting and can result in atrophic sequelae and residual dyschromia (areas of hypo- or hyperpigmentation). Given its similarity to several common conditions, the differential diagnosis includes eczema, psoriasis, fungal infections, chondrodermatitis nodularis, and basal cell carcinoma, which may closely mimic its clinical appearance [1].

To navigate these diagnostic challenges, dermoscopy has emerged as a valuable noninvasive diagnostic tool, enabling the identification of characteristic findings, including whitish-yellow hyperkeratotic follicles, perifollicular whitish halos, follicular plugs, and variable vascular patterns depending on lesion activity. Early recognition of these clinical and dermoscopic features is essential to establish a timely diagnosis, distinguish DLE from other auricular dermatoses, and prevent irreversible scarring and pigmentary sequelae [5].

Therefore, the purpose of this report is to emphasize the diagnostic value of the "carpet tack sign" and dermoscopic findings in identifying discoid lupus at this unusual anatomical site.

## Case presentation

We present the case of a 35-year-old female patient, an elementary school teacher, with a 15-year history of asymptomatic lesions involving the bilateral auricular conchae. She had previously received topical treatment with nystatin cream for one month, without clinical improvement. She had been evaluated at her health care provider, where a potassium hydroxide (KOH) examination of the right conchae was performed and was negative for fungal elements. She denied any history of trauma.

**Figure 1:**
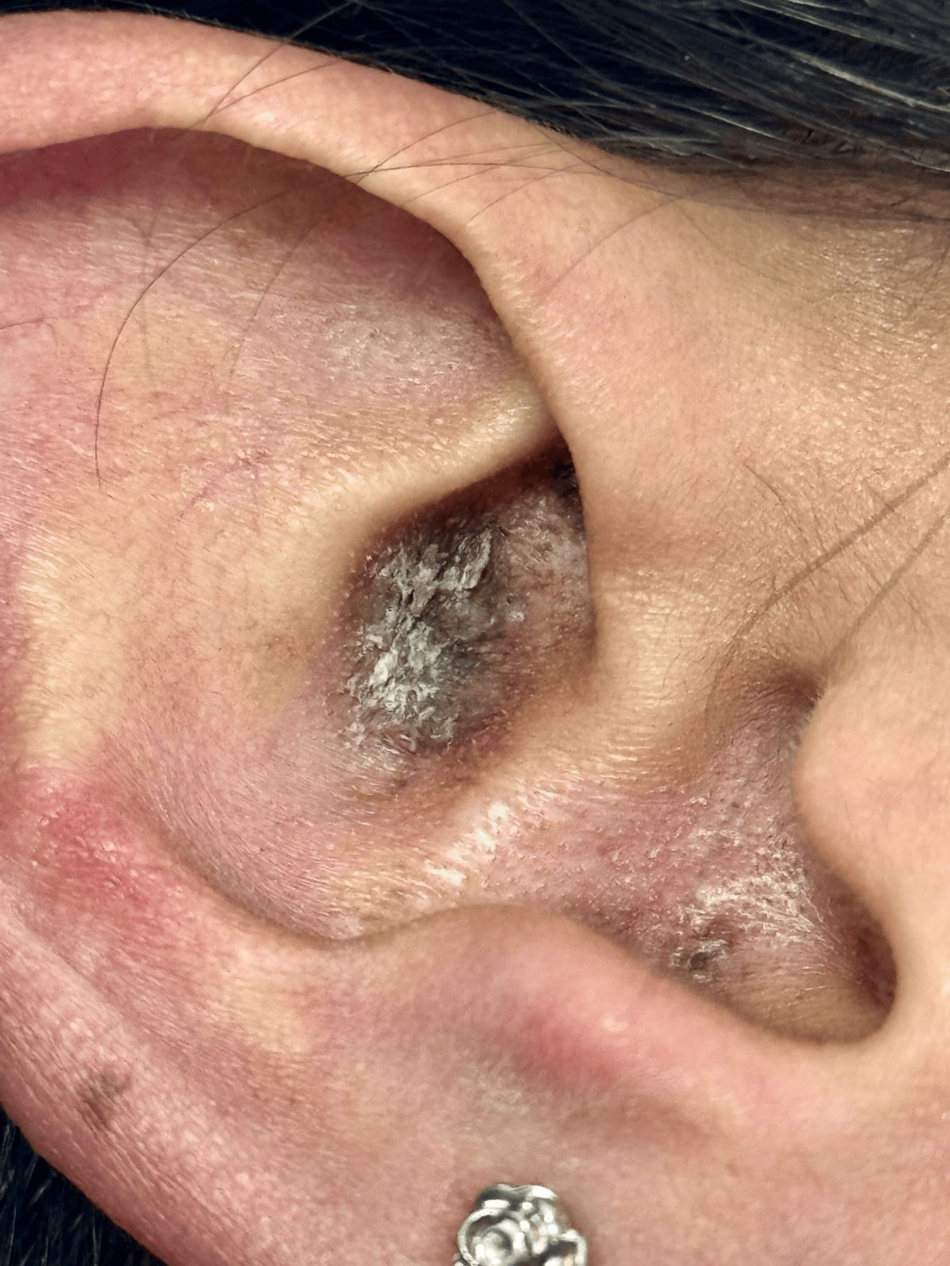
Clinical presentation of auricular discoid lupus erythematosus. Well-demarcated oval violaceous plaques involving the triangular fossa and concha of the right auricle. The lesions exhibit irregular borders associated with thick, dry, adherent whitish scales in a lamellar pattern. Note the presence of whitish filiform keratotic projections (corresponding to the clinical "carpet tack sign") within the follicular orifices.

Her past medical history was unremarkable, except for contact dermatitis triggered by detergents, dust, and paper. On physical examination, oval violaceous plaques with irregular, ill-defined borders were observed on the triangular fossa and bilateral auricular conchae. The lesions exhibited thick, dry, whitish adherent scale arranged in a lamellar pattern. In addition, multiple whitish filiform projections corresponding to the carpet tack sign were noted (Figure 1).

**Figure 2:**
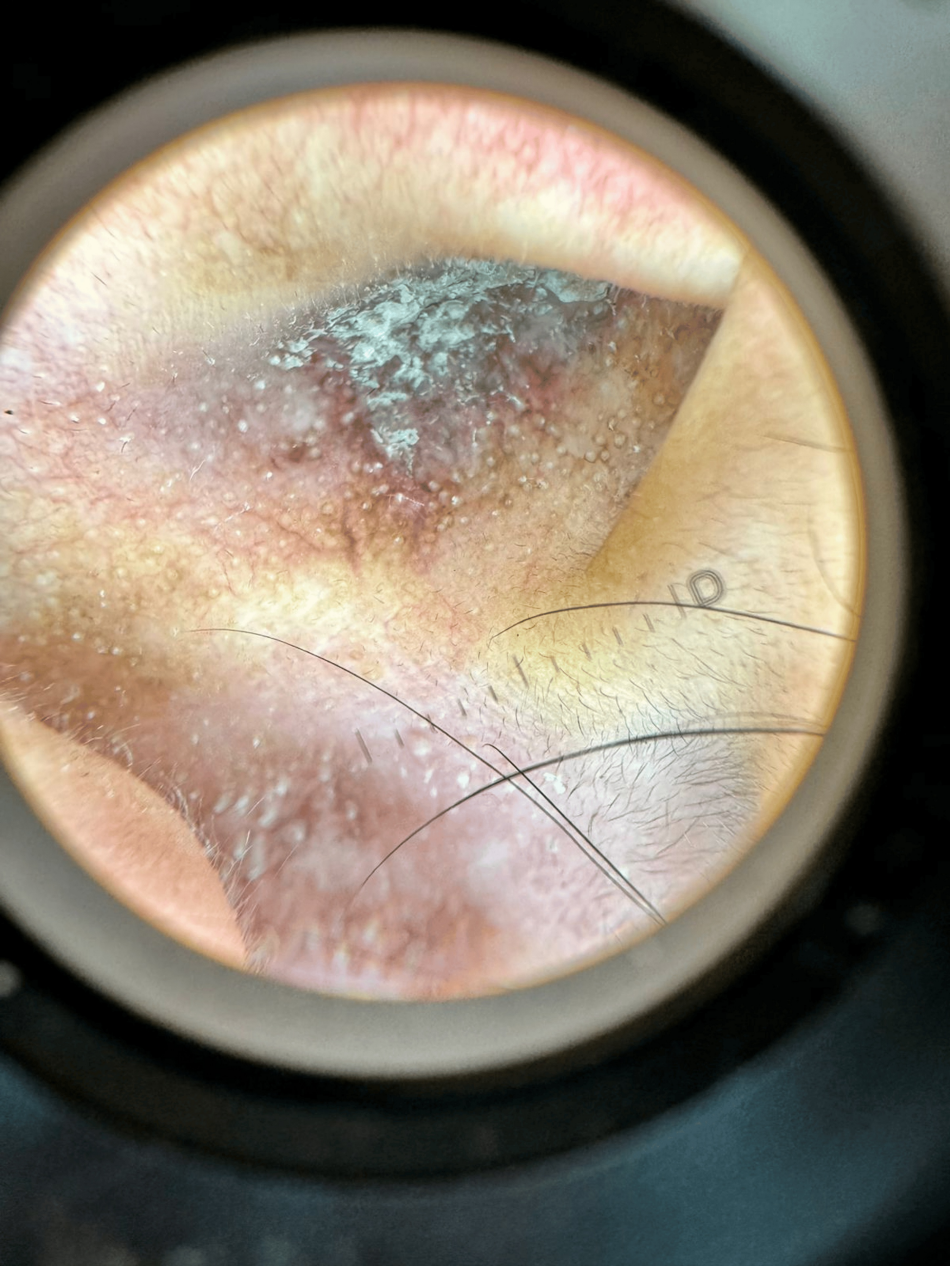
Dermoscopic findings of auricular DLE (right auricle, original magnification 10x). Dermoscopy reveals a brownish-violaceous background with prominent, thick, adherent whitish hyperkeratosis. Multiple whitish pinpoint projections, follicular plugs, are distributed throughout the lesion, surrounded by characteristic perifollicular whitish halos. These findings are highly suggestive of follicular involvement and chronic inflammation. DLE: discoid lupus erythematosus

Dermoscopy revealed brownish-violaceous plaques with thick, adherent whitish scale, multiple whitish pinpoint projections, and perifollicular whitish halos (Figure 2).

**Figure 3:**
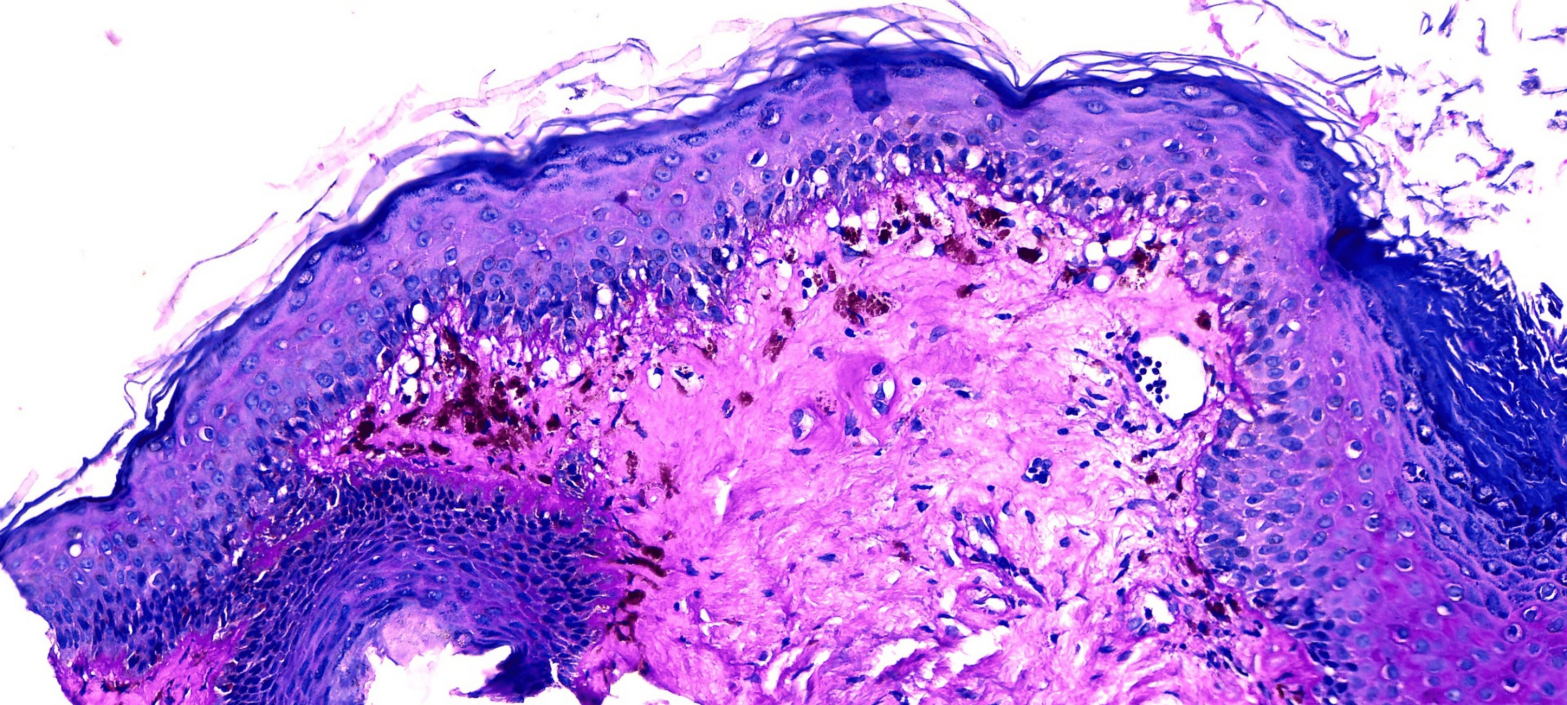
Histopathological analysis of the auricular lesion (Hematoxylin & Eosin stain). The specimen shows characteristic features of discoid lupus erythematosus, including marked hyperkeratosis with prominent follicular plugging. At the dermoepidermal junction, there is a vacuolar interface dermatitis with necrotic keratinocytes. A dense superficial and deep perivascular and periadnexal lymphocytic infiltrate is also observed, supporting the clinical and dermoscopic diagnosis.

A biopsy specimen was obtained from the left auricular concha. Histopathological examination revealed epidermal atrophy with follicular keratotic plugs and interface changes characterized by apoptotic keratinocytes descending into the papillary dermis as hyaline (Civatte) bodies, accompanied by melanophages and mild collagen condensation, along with very sparse chronic inflammatory infiltrate. Periodic acid-Schiff (PAS) and Alcian blue staining demonstrated thickening of the basement membrane and increased interstitial mucin. These findings were consistent with a diagnosis of discoid lupus erythematosus. Representative histopathological images are shown in Figure 3.

Localized auricular DLE poses a diagnostic challenge due to its substantial overlap with inflammatory, infectious, and neoplastic conditions. In our case, chronic cutaneous fungal infection was initially considered; however, a negative potassium hydroxide exam and the presence of scarring excluded this possibility. Psoriasis was deemed unlikely given the absence of silvery micaceous scaling and the presence of follicular plugging with atrophic changes, findings more characteristic of DLE.

Lichen planopilaris and cutaneous sarcoidosis were also part of the differential diagnosis. Nevertheless, the “carpet tack sign” reflecting adherent follicular keratotic plugs is not a feature of these conditions. Lichen planopilaris typically presents with perifollicular erythema and scaling without prominent keratotic plugging, whereas sarcoidosis demonstrates translucent orange-yellow areas on dermoscopy, “apple-jelly” sign rather than follicular hyperkeratosis.

Chronic lesions affecting the auricular region also require careful evaluation to rule out discoid appearing squamous cell carcinoma and basal cell carcinoma. Nevertheless, these neoplastic conditions do not demonstrate the widespread follicular involvement characteristic of the disease. In this case, the presence of firmly adherent follicular keratotic plugs, together with histopathologic evidence of basement membrane thickening and increased dermal mucin, established the diagnosis with certainty.

Following diagnosis, treatment with high-potency topical corticosteroids was initiated on the auricular lesions. At follow-up, the plaques persisted but showed partial clinical improvement, with a reduction in scale thickness and erythema.

During follow-up evaluation, the patient additionally reported hair loss, photosensitivity, left knee pain, morning stiffness in the hands, and intermittent digital edema that improved throughout the day. Given these new systemic symptoms, a laboratory workup was performed. Complement levels (C3 and C4) were within normal limits. Antinuclear antibodies (ANA) were positive, anti-double-stranded DNA antibodies were negative, anti-Ro antibodies were negative, anti-Smith antibodies were positive, and anti-cyclic citrullinated peptide antibodies were negative.

Although complement levels were normal and anti-double-stranded DNA (anti-dsDNA) antibodies were negative, the presence of positive ANA and anti-Smith antibodies, together with photosensitivity and inflammatory articular symptoms, raised concern for possible systemic involvement. The patient was therefore referred for rheumatologic evaluation and ongoing systemic monitoring to assess for progression to systemic lupus erythematosus. Given the presence of systemic symptoms and positive serologic findings, the validated 2019 European League Against Rheumatism (EULAR)/American College of Rheumatology (ACR) classification criteria for systemic lupus erythematosus (SLE) were applied [2,6]. Although the patient fulfilled the entry criterion with a positive antinuclear antibody (ANA) test and had anti-Smith antibodies and musculoskeletal symptoms, the cumulative score did not reach the total required for SLE classification. Therefore, SLE could not be established, and the patient was considered to have cutaneous lupus erythematosus with possible early systemic involvement, warranting close follow-up [7].

## Discussion

Discoid lupus erythematosus (DLE) is the most prevalent form of chronic cutaneous lupus erythematosus. It is characterized by well-demarcated, hyperkeratotic, chronic inflammatory plaques that may progress to atrophy and scarring. The classification of DLE into localized and generalized forms has important prognostic implications; localized disease confined to the head and neck generally follows a more benign, skin-limited course, whereas generalized disease involving areas above and below the neck has been associated with a higher likelihood of progression to systemic lupus erythematosus (SLE). Reported progression rates from DLE to SLE range from approximately 5-10% in cohorts of patients with localized disease to up to 25-30% overall in large observational and systematic review samples, with risk factors including disseminated lesions and positive antinuclear antibodies [1,2,8-10]. In this context, accurate identification of the distribution pattern is essential to determine the individual risk of systemic evolution and to define the appropriate frequency of clinical and serologic follow-up [11].

The pathogenesis of DLE is complex and multifactorial, involving immunologic, genetic, and environmental mechanisms. From an immunologic standpoint, autoantibody formation and immune complex deposition at the dermoepidermal junction lead to persistent inflammation and tissue damage, while complement activation further amplifies the inflammatory response [3,4]. Genetic susceptibility also plays a significant role, with multiple genes involved in immune regulation and antigen recognition predisposing individuals to disease development. These predisposing factors interact with environmental triggers, particularly ultraviolet radiation (UVR) exposure, which promotes keratinocyte apoptosis and autoantigen externalization, thereby initiating or exacerbating cutaneous lesions. UVR is considered the principal environmental precipitant of disease flares and lesion development. Additional factors, such as cigarette smoking - associated with increased disease activity and refractoriness to therapy - and psychological stress have also been identified as important modulators of disease activity in cohort studies [1,3,6]. Recognition of these elements is essential for establishing preventive strategies and optimizing patient education to reduce lesion activity and recurrence.

In the present case, lesion morphology and the presence of the “carpet-tack” sign provided key diagnostic clues. This sign is observed when careful removal of the adherent scale from an active discoid lesion reveals multiple keratotic plugs attached to its undersurface, resembling the tacks used to secure carpets [1,3]. This phenomenon reflects hallmark histopathologic changes of DLE, including follicular hyperkeratosis, dilation of follicular ostia, and deep keratotic plugging, which firmly anchor the scale to the lesion [4,5]. Although not pathognomonic and lacking specific sensitivity and specificity studies in the current literature, its diagnostic utility stems from its direct correlation with follicular plugging, a histopathological hallmark of DLE. According to dermoscopic studies by Lallas et al. and Errichetti et al., the identification of these follicular keratotic structures serves as a highly suggestive feature that helps distinguish DLE from other scarring and inflammatory dermatoses, such as lichen planopilaris or scalp psoriasis, thereby supporting its role as a key clinical indicator in the diagnostic workup [5,12].

Complementing the physical examination, dermoscopy commonly demonstrates follicular keratotic plugs, perifollicular whitish halos, structureless white areas corresponding to dermal fibrosis, and a mixed vascular pattern composed of linear and branching vessels on an erythematous background [4,5]. Histopathology remains essential to confirm the diagnosis, revealing hyperkeratosis with compact orthokeratosis, prominent follicular plugging, focal epidermal atrophy, vacuolar interface dermatitis, and thickening of the basement membrane. In the dermis, typical features include superficial and deep perivascular and periadnexal lymphocytic infiltrates, increased dermal mucin, pigment incontinence with melanophages in the papillary dermis, and fibrosis in chronic lesions. Direct immunofluorescence further supports the diagnosis by demonstrating the classic lupus band - granular deposits of immunoglobulins (IgG, IgM, IgA) and complement (C3) along the dermoepidermal junction - detectable in lesional skin and occasionally in clinically uninvolved skin; this finding significantly strengthens the suspicion of DLE [3,4]. Although direct immunofluorescence (DIF) is a useful adjunct in the diagnostic evaluation of discoid lupus erythematosus, it was not performed in our case. The diagnosis was established based on the concordance of clinical features, dermoscopic findings, and histopathological changes, which are generally considered sufficient in typical presentations [1,6]. In addition, DIF has variable sensitivity and may yield false-negative results, particularly in long-standing or previously treated lesions [1,6]. Given the chronicity of the lesions and the clear clinicopathologic correlation, the procedure was not considered essential for diagnostic confirmation in this patient.

Treatment of DLE aims to prevent lesion progression and scarring, limit scarring alopecia, and reduce the risk of progression to SLE through a combination of strict photoprotection, general measures, and topical or systemic therapies [1,4,6,11]. Photoprotection is the cornerstone of management and includes daily use of broad-spectrum sunscreens (SPF ≥50), protective clothing, sun avoidance, and modification of risk factors such as smoking and the use of photosensitizing medications. In localized disease, high-potency topical corticosteroids and calcineurin inhibitors are first-line treatments. In disseminated or refractory forms, antimalarial agents such as hydroxychloroquine, chloroquine, or quinacrine are considered. In severe, hypertrophic, or treatment-resistant cases, second-line agents including thalidomide, dapsone, systemic retinoids, or immunosuppressants such as methotrexate, mycophenolate mofetil, or azathioprine may be used. In exceptional situations, biologic agents have been employed off-label in severely refractory patients. Physical therapies, such as intralesional corticosteroids, cryotherapy, or laser treatment, may be useful for hypertrophic lesions or aesthetic sequelae. At the same time, hair transplantation is reserved for stable scarring alopecia in inactive disease [6,10]. In the present case, high-potency topical corticosteroids were initiated due to the localized distribution of the lesions and the absence of confirmed systemic disease at diagnosis. However, chronic, bilateral, and long-standing lesions, particularly in cosmetically sensitive areas such as the auricle, may benefit from earlier consideration of systemic therapy to prevent progression and irreversible scarring. Antimalarial agents, especially hydroxychloroquine, represent first-line systemic therapy in cutaneous lupus erythematosus and may be considered even in localized cases with risk factors for persistence or progression [6,9]. In retrospect, the clinical features of our patient suggest that earlier systemic therapy could have been considered, underscoring the importance of individualized management and close follow-up.

## Conclusions

This case highlights the clinical value of the “carpet-tack” sign as a useful semiological marker in the diagnosis of localized discoid lupus erythematosus, particularly in uncommon sites such as the auricular concha. However, its diagnostic utility may be limited in early or minimally hyperkeratotic lesions and in previously treated plaques, where follicular plugging and adherent scaling may be less evident.

Therefore, recognition of this sign should be integrated with dermoscopic evaluation and histopathologic correlation to enhance diagnostic accuracy. Dermoscopic features, including follicular keratotic plugs, perifollicular whitish halos, and characteristic vascular patterns, complement the clinical assessment and aid in differentiating DLE from other inflammatory dermatoses.

Early identification through this combined approach facilitates timely treatment and may reduce the risk of permanent scarring; nonetheless, further prospective studies are needed to establish the specific sensitivity, specificity, and prognostic significance of these findings in auricular presentations.
